# Remarkably and stable catalytic activity in reduction of 4-nitrophenol by sodium sesquicarbonate-supporting Fe_2_O_3_@Pt†

**DOI:** 10.1039/d3ra01930f

**Published:** 2023-05-03

**Authors:** Xia Xu, Mingqiang Li, Liming Yang, Bing Hu

**Affiliations:** a College of Science, Gansu Agricultural University Lanzhou 730070 P. R. China xuxia@gsau.edu.cn; b College of Chemistry, Xinjiang University Urumqi Xinjiang 830046 P. R. China

## Abstract

Reasonable design of bimetallic nanomaterials with support is beneficial to improve catalytic performance. This work reports a new kind of sodium sesquicarbonate-supporting Fe_2_O_3_@Pt *via* etching Fe_3_O_4_@Pt@SiO_2_, which exhibits highly efficient and stable catalytic reduction performance towards 4-NP. Sodium sesquicarbonate-supporting Fe_2_O_3_@Pt has an interconnected one-dimensional network structure that provides sufficient channels for mass transfer. At the same time, a large amount of Fe_2_O_3_@Pt is exposed on its surface, which hinders the aggregation of pt clusters and Fe_2_O_3_ nanoparticles, and facilitates the direct contact of Fe_2_O_3_@Pt reaction sites with reactant molecules, thus improving the catalytic rate of 4-NP reduction reaction. Moreover, the introduction of non-metallic Fe can not only reduce the consumption of precious metal Pt, but also improve the catalytic efficiency due to the synergistic effect. This study opens up a new avenue to develop robust catalysts for heterogeneous catalytic reactions.

## Abstract 2

The sodium sesquicarbonate-supporting Fe_2_O_3_@Pt can not only prevent the aggregation of pt clusters and Fe_2_O_3_ nanoparticles, but also ensure the synergistic effect between Pt and Fe, and improve the catalytic activity for 4-NP reduction.

## Graphical abstract

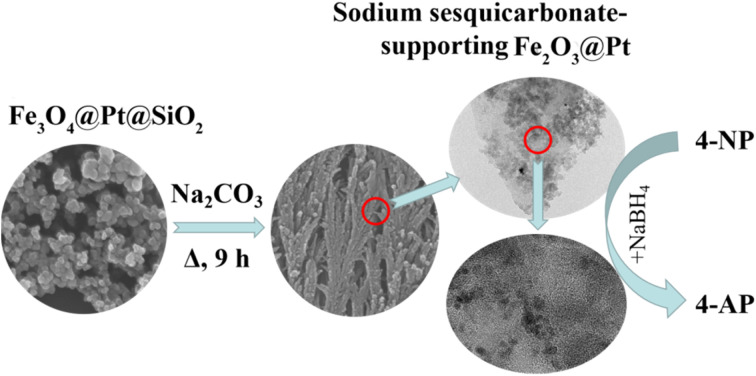

## Introduction

In recent decades, environmental pollution and the energy crisis are threatening human life, leading to a variety of deteriorative consequences. Nitrophenol and its derivatives are some of the most refractory pollutants that occur in industrial wastewater, synthetic dyes, pharmaceuticals and other industries.^1,2^ Among them, 4-nitrophenol (4-NP) is regarded by the U.S. government as one of the toxic and refractory priority pollutants endangering the human central nervous system.^3–5^ Nevertheless, aminophenol with low toxicity is an important chemical intermediate in the synthesis of drugs, dyes, pesticides and imaging agents.^6^ Therefore, it is of great environmental and energy significance to eliminate nitro groups in toxic phenolic compounds or convert them into amino groups. Additionally, the conversion of nitrophenol to aminophenol has considerable industrial relevance for aniline and paracetamol production.^7^

Among various methods, the reduction of 4-nitrophenol (4-NP) to 4-aminophenol (4-AP) using reducing agents (such as NaBH_4_) is one of the simplest and cost-effective methods.^8,9^ This process requires *E*_0_ (4-NP/4-AP) = −0.76 V, which is thermodynamically appropriate, but there is a high kinetic barrier among the 4-NP and BH_4_^−^ ions. Therefore, it is particularly important to select a suitable catalyst for the catalytic reduction of 4-NP to 4-AP. Noble metal nanoparticles have attracted attention due to their distinctive physicochemical properties.^10–14^ The catalytic reduction of 4-NP by noble metal nanostructures has been extensively investigated, including Au,^15,16^ Pd,^17,18^ Ag,^19,20^ Pt,^21,22^ Ag-Pt,^23^ Au-Pt,^24^*etc.* These catalysts exhibit good catalytic reduction effect toward 4-NP. However, noble metal nanoparticles have small size, large specific surface area and high specific surface energy, so there is a trend of aggregation, which will lead to the gradual decline of the catalytic activity. Therefore, insufficient catalyst utilization coefficient, low mass transfer rate and unstable deactivation of noble metal catalysts are still some common problems that limit their application.^25^

To maximize the catalytic activity of noble metal nanoparticles, it is necessary to choose appropriate carrier materials to disperse them. Some oxides are used as carriers to disperse noble metal nanoparticles, such as Fe_2_O_3_,^26^ Al_2_O_3_,^27^ SiO_2_,^28^ and CeO_2_.^29^ As the carrier of noble metal nanoparticles, two-dimensional membrane materials have more advantages, which can not only improve the catalytic activity, but also improve the stability of the catalyst. Zhang's group loaded Au–Pt alloy nanoparticles on α-Co(OH)_2_ nanosheets to improve the catalytic performance for the 4-NP reduction reaction.^30^ Han's group used Pd nanospheres decorated reduced graphene oxide for highly efficient catalytic reduction of the hazardous 4-nitrophenol pollutant.^31^ Our research group has also published a study on the high efficiency reduction of *p*-nitrophenol (4-NP) by Au atomic cluster decorated Fe(OH)_3_/Fe_2_O_3_ nanosheets.^32^ In addition, relevant literature reports that inorganic salt materials can also be used as carriers. Yang's group describe the synthesis of Au nanoparticles coated chicken eggshell composites (defined as Au/CaCO_3_ nanocomposite), which can also be used as a sensor and catalyst for the efficient detection and treatment of 4-NP.^33^

On the other hand, due to the high cost and scarcity of noble metals, their wide application has been severely hampered. Relevant literature reports that transition metals can also be used as catalysts for the catalytic reduction of 4-NP. CuFe_2_O_4_ magnetic nanoparticles prepared by Chen's group exhibited several advantages such as stability, monodispersity, low-cost, simplicity and rapid separation performance over other catalysts for the reduction of nitrophenol.^34^ Chiaki Terashima's group use nanoflakes-like NiCo_2_O_4_ samples as catalysts for rapid reduction of 4-nitrophenol.^35^ In addition, Cu/graphdiyne material prepared by Li's group also exhibits high catalytic ability for rapid reduction of 4-NP within 3 min.^36^ These literature show that the transition metal nanomaterials also have a good catalytic effect on the 4-NP reduction reaction.

Furthermore, the reasonable design of bimetallic catalysts, based on alloying of noble metal with other transition metal (such as Cu, Co), is not only crucial to reduce costs, but even more importantly, to improve the physical and chemical properties of alloys.^37^ Li's group reported a clean and composition-tunable PdCu and AuCu monolithic aerogels with 3D nanowire-based network for enhanced catalytic reduction of 4-nitrophenol, the introduction of nonprecious Cu not only drastically cuts down the cost but also attains the excellent catalytic activity due to the bimetallic intrinsic synergetic effect.^38,39^ However, there are few reports on the catalytic reduction of 4-NP by bimetallic catalysts based on the alloying of noble metals with other easily oxidized transition metals.

Herein, Fe_3_O_4_@Pt were prepared. Although the synergistic effect between Pt and Fe greatly improves the catalytic rate for 4-NP reduction reactions, Fe_3_O_4_/Pt is easily separated and aggregated during use or storage, resulting in a worsening catalytic rate. Therefore, Fe_2_O_3_@Pt was designed to be loaded onto the surface of sodium sesquicarbonate crystals. The prepared sodium sesquicarbonate structure can not only prevent the aggregation of Pt cluster and Fe_2_O_3_ nanoparticle, but also ensure the synergistic effect between Pt and Fe, and improve the catalytic activity for 4-NP reduction.

## Experimental

### Materials

Chloroplatinic acid hexahydrate (H_2_PtCl_6_·6H_2_O), 2-morpholinoethanesulfonic acid (MES), hexadecyl trimethyl ammonium bromide (CTAB), polyvinylpyrrolidone (PVP) and *p*-nitrophenol (4-NP) were obtained from Aladdin reagent (Shanghai) Co., Ltd. Tetraethyl orthosilicate (TEOS) were obtained from Shanghai Zhongqin Chemical Reagent Co., Ltd. Ethanol and aqueous ammonia were obtained from Beijing Chemical Reagent. FeSO_4_ and Na_2_CO_3_ were obtained from Sinopharm Chemical Reagent Co., Ltd. NaBH_4_ was obtained from Jinan Yuxin Chemical Co., Ltd. All reagents used in this study are analytically pure and have not been purified in any way except for special labels. All experiments used deionized water.

### Synthesis of Fe_3_O_4_@Pt

First, 5 mL distilled water and 4 mg FeSO_4_ were added into a 50 mL beaker for ultrasonic dispersion. Then add 5 mL ethanol and 0.2 mL H_2_PtCl_6_ aqueous solution (1 g/100 mL). After fully stirring, 5 mg NaBH_4_ was added into the mixed system. Centrifuge after stirring for ten minutes, discard the supernatant, wash the sediment with distilled water before dispersing the sediment in 2 mL distilled water for standby.

### Synthesis of Fe_3_O_4_@Pt@SiO_2_

First, 5 mL distilled water, 4 mg FeSO_4_ and 100 mg MES were added into a 50 mL beaker for ultrasonic dispersion. Then add 5 mL ethanol, 0.2 mL H_2_PtCl_6_ aqueous solution (1 g/100 mL) and 45 μL TEOS. After fully stirring, 5 mg NaBH_4_ and 0.7 mL ammonia water were added into the mixed system at the same time. Centrifuge after stirring for ten minutes, discard the supernatant and wash the sediment with distilled water. Mark the prepared sample as Fe_3_O_4_@Pt@SiO_2_.

The synthesis method of the Fe_3_O_4_@Pt@SiO_2_ with different experimental condition was the same as the above, except that without MES or with 100 mg MES and 100 mg PVP or with 100 mg MES and 100 mg CTAB.

### Synthesis of sodium sesquicarbonate-supporting Fe_2_O_3_@Pt

Wheat spike shaped sodium sesquicarbonate-supporting Fe_2_O_3_@Pt could be obtained by etching the Fe_3_O_4_@Pt@SiO_2_. The etching method is as follows: Fe_3_O_4_@Pt@SiO_2_ obtained by the above method was ultrasonically dispersed in 10 mL distilled water. Then add 0.035 g CTAB and 1.06 g Na_2_CO_3_ successively, and stir in a constant temperature oil bath at 80 °C. 100 μL mixed solution was taken from the system every 3 hours to monitor the change of catalytic performance. Centrifuge after etching for 9 hours. The product was centrifuged at hot time, and washed with hot water five times, then dispersed in 2 mL distilled water for standby.

### The catalytic performance test for 4-NP reduction reaction

The catalytic performance test was carried out at room temperature with a 4 mL quartz colorimetric dish as the reaction vessel. The absorbance performance during reaction was monitored by 722N visible spectrophotometer or UV-vis spectra. The specific operation steps are as follows: 3 mL H_2_O, 20 μL 4-NP (0.01 mol L^−1^), and 20 μL catalyst solution (Pt nanoparticles, Fe_3_O_4_ nanoparticles or sesquicarbonate-supporting Fe_2_O_3_@Pt solution take from 2 mL spare solution) were successively added into a 4 mL quartz colorimetric dish. After the solution was completely dispersed evenly, 100 μL fresh NaBH_4_ (0.2 mol L^−1^) aqueous solution is dropped into the above solution. Then continue to record the absorbance value at *λ* = 400 nm or scan the UV-vis spectrum in the range of 250–500 nm immediately.

### Characterizations

The powder X-ray diffraction (XRD) patterns were performed on a Panaco X′ Pert PRO X-ray diffractometer with Cu Kα radiation (*l* = 0.15406 nm) with the operation voltage and current maintained at 45 kV and 40 mA. Scanning speed: 1° min^−1^, test range: 10° ≤ 2*θ* ≤ 70°. Fourier transform infrared spectroscopy (FT-IR) analysis was carried out on a PerkinElmer FT-IR spectrometer. The scanning electron microscope (SEM) images were determined by Zeiss MERLIN Compact SEM-EDX microanalysis. TEM images and elemental mapping analysis of the samples were obtained on a FEI Talos F200S transmission electron microscope operating at 200 kV. The chemical states of different elements were determined by Thermo ESCALAB 250XI X-ray photoelectron spectroscopy (XPS). The UV-vis absorption spectra of the samples were measured on Shimadzu UV-1780 UV-vis-NIR spectrophotometer. The absorbance of the solution during the reaction was monitored by a 722N visible spectrophotometer.

## Results and discussions

### The catalytic performance of Fe_3_O_4_@Pt for 4-NP reduction reaction

4-NP solution has an obvious absorption peak at 400 nm. The reduction of 4-NP to 4-AP using reducing agents NaBH_4_ is thermodynamically appropriate, but there is a high kinetic barrier among the 4-NP and BH_4_^−^ ions. Therefore, precious metal nanoparticles are the first choice for catalytic reduction of 4-NP. Fig. S1† shows the reaction process and time-dependent absorption spectrum of the reaction mixture in the wavelength range of 250–500 nm for the catalytic reduction of 4-NP by Pt nanoparticles at room temperature. The decrease in the 400 nm peak indicates the disappearance of 4-NP, whereas the formation of a new peak at 300 nm indicates the formation of 4-AP. Therefore, the absorbance value A at 400 nm could represent the concentration C of 4-NP in the solution. Since the amount of NaBH_4_ is excessive compared with that of 4-NP, the reaction kinetics follows the pseudo-first-order law, the apparent kinetic rate constant *k*_app_ can be obtained according to eqn (1)1
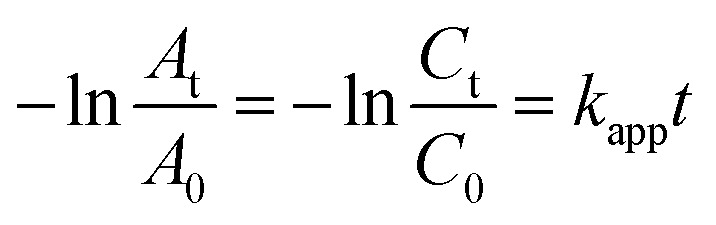
where *t* is the reaction time, *A*_t_ and *A*_0_ stand for the absorbance at time *t* and the initial absorbance of 4-NP, *C*_t_ and *C*_0_ is the concentration at time *t* and the initial concentration of 4-NP.

**Fig. 1:**
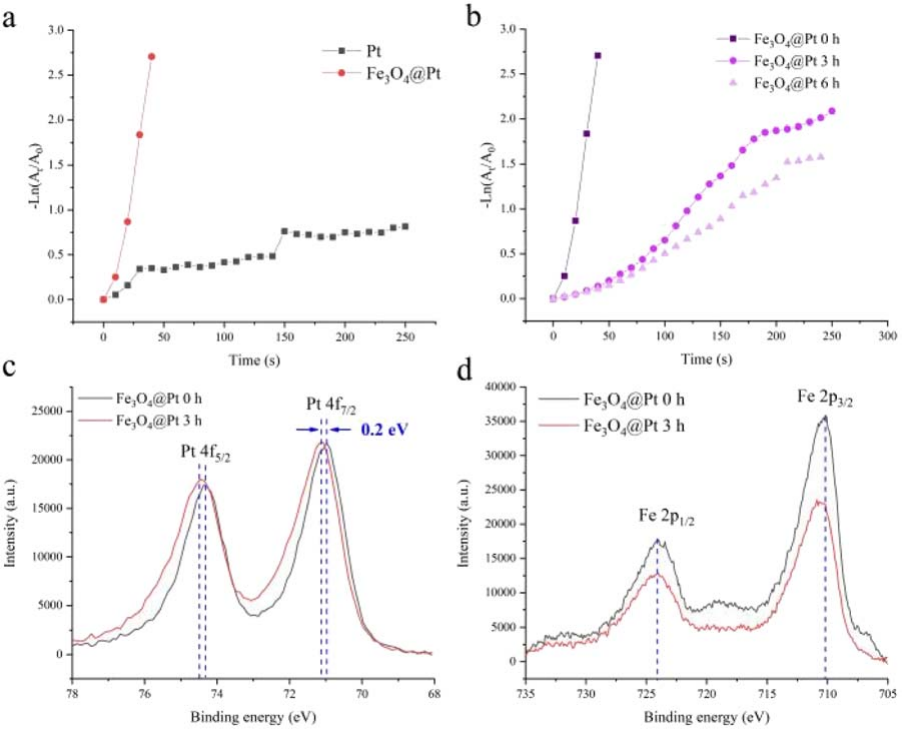
Plots of −ln(*A*_t_/*A*_0_) *versus* the reaction time *t* for the reduction of 4-NP catalyzed by: (a) Pt and Fe_3_O_4_@Pt, (b) Fe_3_O_4_@Pt 0 h, 3 h, and 6 h, as well as the XPS fine spectra of (c) Pt 4f, (d) Fe 2p.

It can be seen from the Fig. 1a that the catalytic rate of Pt nanoparticles is not very ideal. In order to reduce the consumption of precious metals and solve the problems of insufficient utilization coefficient, low mass transfer rate and unstable deactivation, Fe_3_O_4_@Pt were designed and prepared. Fig. S2† shows the XRD result of Fe_3_O_4_@Pt, the diffraction peak at about 40° is attributed to Pt nanoparticles. The fine XPS spectrum of Fe element (as shown in Fig. S3†) indicates that Fe exists in the form of divalent and trivalent. The catalytic reduction effect of Fe_3_O_4_@Pt on 4-NP reduction reaction is much better than that of Pt, and Fe_3_O_4_ nanoparticles has no catalytic reduction effect (Fig. S4†). Therefore, it is speculated that the excellent catalytic performance of Fe_3_O_4_@Pt on the reduction of 4-NP was attributed to the synergistic effect between Pt and Fe. However, with the extension of the sample storage time, the catalytic performance of Fe_3_O_4_@Pt gradually weakened. When stored in aqueous solution for 3 hours, its catalytic rate decreases to half of the original one, and becomes worse after 6 hours.

**Fig. 2:**
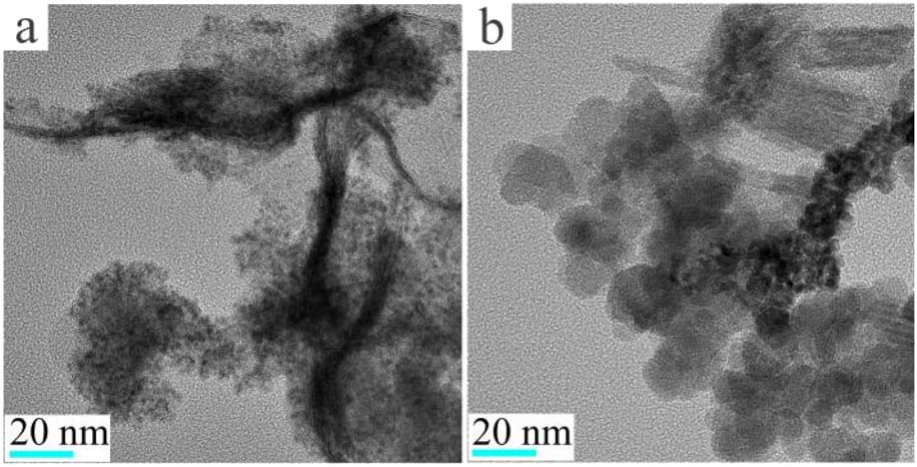
The TEM images of (a) Fe_3_O_4_@Pt 0 h and (b) Fe_3_O_4_@Pt 3 h at same magnifications.

The reasons for the poor catalytic effect of Fe_3_O_4_@Pt were characterized by XPS and TEM. As shown in Fig. 1c, the high-resolution Pt 4f peaks near the binding energies of 71.0 and 74.5 eV are attributed to Pt 4f_7/2_ and 4f_5/2_, respectively. Notably, the Fe_3_O_4_@Pt (0 h) peak shows 0.2 eV negative shift compared to the Fe_3_O_4_@Pt (3 h), indicating the charge transfer from Fe to Pt in Fe_3_O_4_@Pt (0 h) and lead to the excellent catalytic effect of Fe_3_O_4_@Pt (0 h).^41,42^ In Fig. 1d, the peak at 710.0 and 724.2 eV were originated from Fe 2p_3/2_ and Fe 2p_1/2_, respectively. The TEM results of Fe_3_O_4_@Pt (0 h) and Fe_3_O_4_@Pt (3 h) were shown in Fig. 2. In Fe_3_O_4_@Pt (0 h), the dispersion and homogeneity of Pt and Fe_3_O_4_ are particularly good. Pt clusters of about 2 nm is evenly distributed on the surface of Fe_3_O_4_. After the sample is dispersed in aqueous solution for 3 h, Pt and Fe_3_O_4_ are separated from each other and agglomerated. Therefore, the interaction between Pt and Fe is reduced, and the catalytic reduction effect becomes worse. Combined with XPS and TEM results, Fe_3_O_4_@Pt (0 h) has a good catalytic effect on 4-NP reduction due to the synergistic effect between Pt and Fe.

### Preparation and characterization of sodium sesquicarbonate-supporting Fe_2_O_3_@Pt

**Fig. 3:**
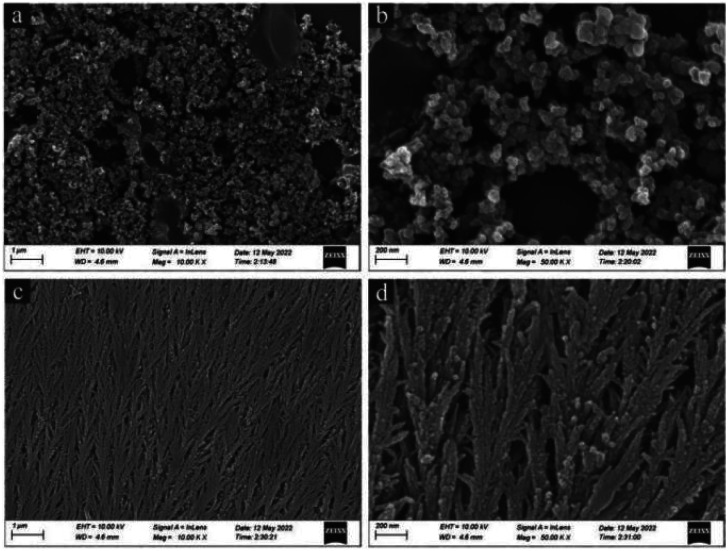
The scanning electron microscope (SEM) images of (a and b) Fe_3_O_4_@Pt@SiO_2_, (c and d) sodium sesquicarbonate-supporting Fe_2_O_3_@Pt at different magnifications.

In order to prevent the separation of Pt and Fe_3_O_4_ and the agglomeration of Pt nanoparticles, newly prepared Fe_3_O_4_@Pt were encapsulated in SiO_2_ nanospheres (labeled as Fe_3_O_4_@Pt@SiO_2_), and then SiO_2_ was etched away by Na_2_CO_3_ to prepare sodium sesquicarbonate-supporting Fe_2_O_3_@Pt. As SEM results shown in Fig. 3, Fe_3_O_4_@Pt@SiO_2_ nanoparticles present irregular polyhedron and adhere to each other, which is attributed to the rapid polymerization of TEOS under alkaline conditions, and the Fe_3_O_4_@Pt nanoparticles are basically encapsulated in SiO_2_ polyhedron. After etching with Na_2_CO_3_ solution at 80 °C for 9 hours, irregular Fe_3_O_4_@Pt@SiO_2_ polyhedron are transformed into wheat ear like one-dimensional materials, as shown in Fig. 3c and d.

**Fig. 4:**
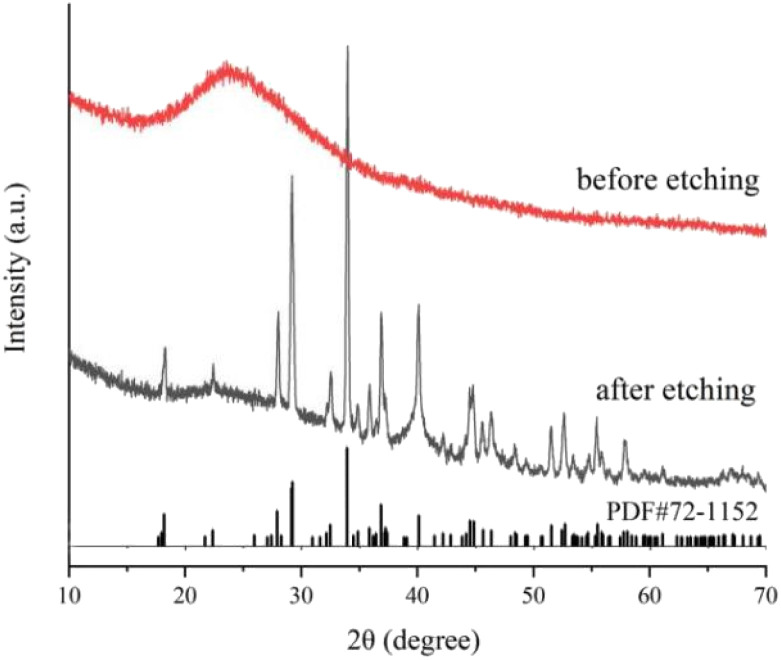
X-ray powder diffraction (XRD) patterns of Fe_3_O_4_@Pt@SiO_2_ (before etching) and wheat spike shaped sodium sesquicarbonate-supporting Fe_2_O_3_@Pt (after etching).

XRD and FT-IR spectra verified the composition, crystal phase and surface characteristics of samples before and after Na_2_CO_3_ etching. According to X-ray diffraction results (Fig. 4), only a strong SiO_2_ (JCPDS#29-0085) diffraction peak at 20–30° is observed before Na_2_CO_3_ etching. However, a series of strong diffraction peaks were observed after Na_2_CO_3_ etching, the diffraction peaks at 18.17°, 27.90°, 29.12°, 29.21°, 33.91°, 36.83° and 40.09° correspond to (400), (111), (402), (3̄11), (5̄11), (800) and (204) crystal planes of sodium sesquicarbonate (JCPDS#72-1152), respectively. The FT-IR spectra also reflect the evolution of surface functional groups before and after etching. As shown in Fig. S5,† before etching, the strong and wide absorption band at 1077 cm^−1^ can be classified as Si–O–Si antisymmetric stretching vibration, and the wide peak at 3128 cm^−1^ is –OH antisymmetric stretching vibration on the SiO_2_ surface. After etching by Na_2_CO_3_, the above two characteristic peaks disappeared, and a new characteristic absorption peak appeared at 1457 cm^−1^, which was attributed to the stretching vibration of CO_3_^2−^. The XRD and FT-IR results are consistent with SEM results. Before etching, Fe_3_O_4_@Pt were encapsulated in SiO_2_, only the diffraction peak of SiO_2_ and the absorption band of Si–O–Si antisymmetric stretching vibration could be detected. And after Na_2_CO_3_ etching, the SiO_2_ disappeared and there was a large amount of CO_3_^2−^ on the surface. Therefore, SEM, XRD and FT-IR spectra confirmed the formation of wheat spike shaped sodium sesquicarbonate crystals.

**Fig. 5:**
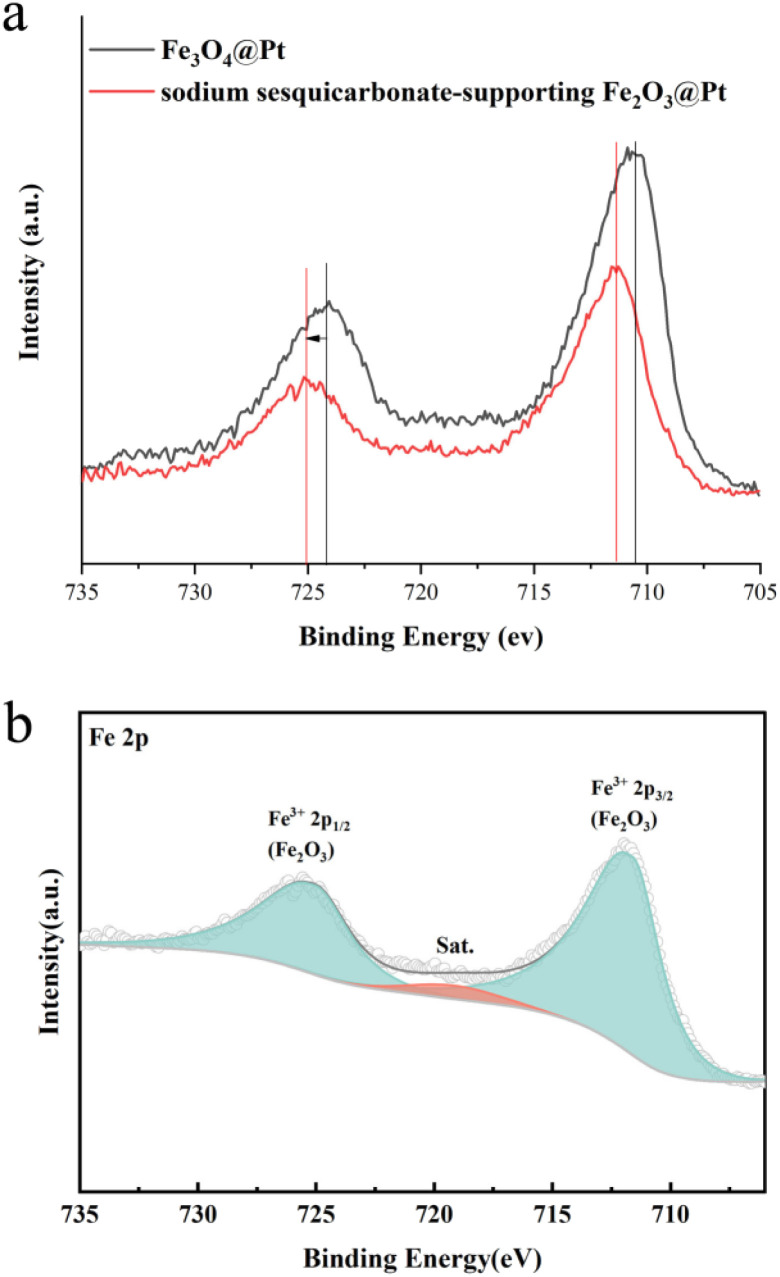
The Fe 2p XPS fine spectra of (a) Fe_3_O_4_@Pt and sodium sesquicarbonate-supporting Fe_2_O_3_@Pt, (b) sodium sesquicarbonate-supporting Fe_2_O_3_@Pt.

Due to the strong crystallinity of sodium sesquicarbonate crystals, the presence of Fe in the etched sample cannot be determined from XRD results. Therefore, the XPS fine spectrum of Fe was characterized. As shown in Fig. 5, the Fe 2p fine spectrum showed a significant shift after etching, and Fe exists in a trivalent form. Therefore, we speculate that Fe_3_O_4_ has transformed into Fe_2_O_3_.

**Fig. 6:**
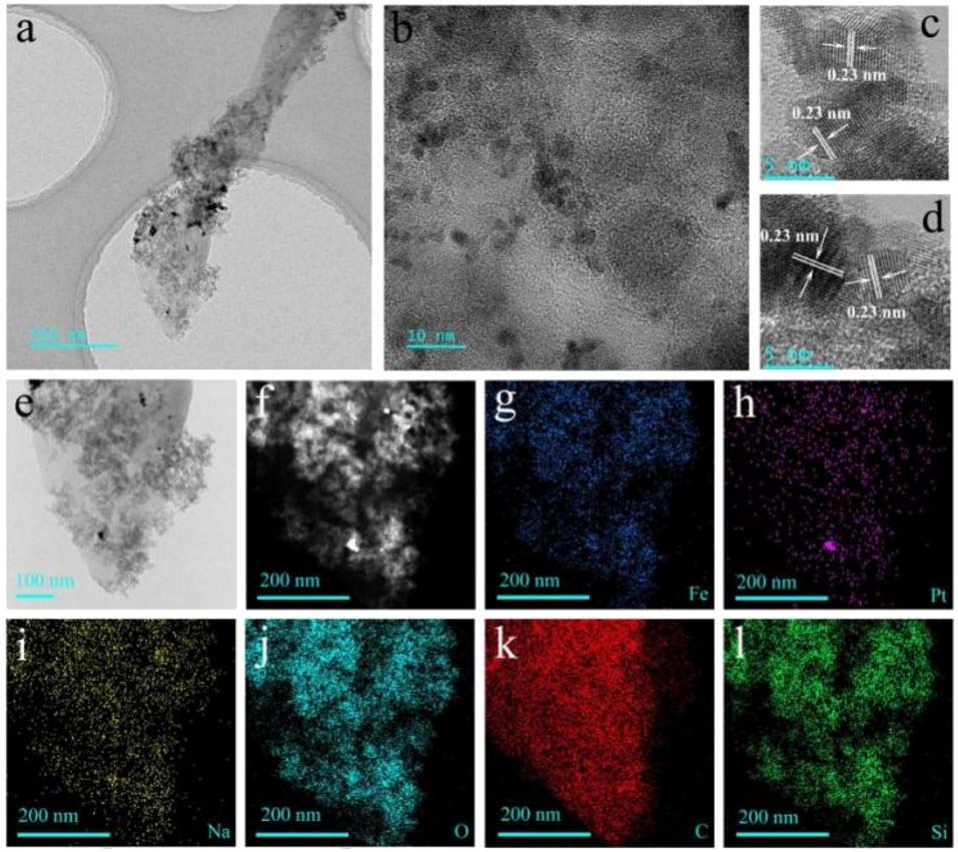
(a–d) TEM image of wheat spike shaped sodium sesquicarbonate-supporting Fe_2_O_3_@Pt, (e–l) corresponding elemental mappings of Fe, Pt, Na, O, C and Si according to the image in (e).

In order to further verify the composition of sodium sesquicarbonate crystal and the distribution of Fe_2_O_3_@Pt, the TEM images and the EDX elemental mapping patterns were characterized. As can be seen from Fig. 6, the residual SiO_2_ after etching is mixed with Fe_2_O_3_@Pt and uniformly adheres to the surface of sodium sesquicarbonate crystal (marked as sodium sesquicarbonate-supporting Fe_2_O_3_@Pt). Pt nanoparticles are uniform in size, with an average diameter of 2–3 nm. Due to natural self-assembly, they are in a disordered state. The HRTEM images of Pt nanoparticles presented all-pervading lattice fringes with a spacing of 0.23 nm, which corresponding to (111) interplane distance of cubic Pt (Fig. 6c and d). The chemical composition of sodium sesquicarbonate-supporting Fe_2_O_3_@Pt was also revealed by energy-dispersive X-ray (EDX) analysis. The EDX spectrum shown in Fig. S6† verified that the as-prepared sodium sesquicarbonate supporting Fe_2_O_3_@Pt is indeed doped with some Si. The EDX elemental mapping patterns further demonstrated the detailed composition distribution of sodium sesquicarbonate-supporting Fe_2_O_3_@Pt, indicating the homogeneous distribution of Pt and Fe elements (Fig. 6e–l).

### Catalytic performance of sodium sesquicarbonate-supporting Fe_2_O_3_@Pt for 4-NP reduction

**Fig. 7:**
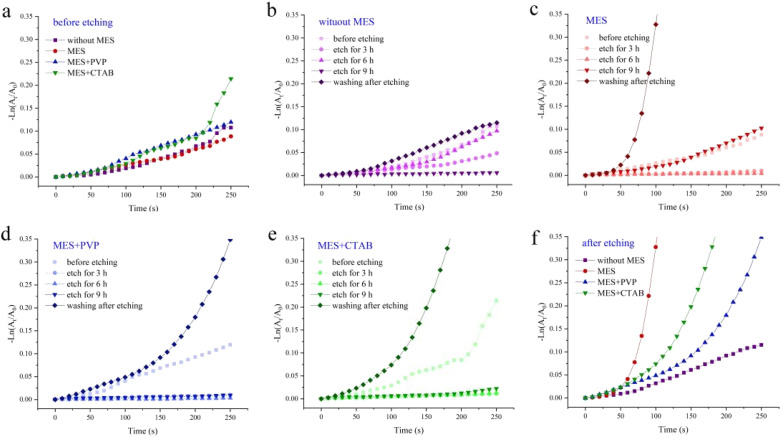
Plots of −ln(*A*_t_/*A*_0_) *versus* the reaction time *t* for the reduction of 4-NP catalyzed by: (a) Fe_3_O_4_@Pt@SiO_2_, (b–f) sodium sesquicarbonate-supporting Fe_2_O_3_@Pt under different preparation conditions.

The catalytic performances of Fe_3_O_4_@Pt@SiO_2_ (before etching) and sodium sesquicarbonate-supporting Fe_2_O_3_@Pt (after etching) on 4-NP reduction reaction with different conditions were investigated. As shown in Fig. 7a, the catalytic activity of Fe_3_O_4_@Pt@SiO_2_ prepared under different conditions (without MES, MES, MES + PVP and MES + CTAB) are similar, and the catalytic rate are very slow due to the encapsulation of SiO_2_. During the etching process (Fig. 7b–e), the catalytic rate becomes slower and slower. When the excess Na_2_CO_3_ is washed away after 9 hours of etching, the catalytic rate is significantly higher than that before etching. It is believed that the rate determining step of the reaction is the reaction between the adsorbed 4-NP and the surface hydrogen species released from NaBH_4_ on the catalyst. Wheat spike shaped sodium sesquicarbonate-supporting Fe_2_O_3_@Pt has an interconnected network structure to provide sufficient channels for mass transfer. At the same time, a large amount of Fe_2_O_3_@Pt is exposed on its surface, which is conducive to the direct contact between Fe_2_O_3_@Pt reaction sites and reactant molecules, thus improving the catalytic rate of 4-NP reduction reaction.

The sodium sesquicarbonate-supporting Fe_2_O_3_@Pt prepared under different conditions (without MES, MES, MES + PVP and MES + CTAB) showed great differences in their catalytic properties (Fig. 7f). Owing to the hindrance of PVP and CTAB, the etched sodium sesquicarbonate-supporting Fe_2_O_3_@Pt with PVP or CTAB showed the lower catalytic rate. However, sodium sesquicarbonate-supporting Fe_2_O_3_@Pt with MES showed the best catalytic performance due to the reduction protection characteristics of MES.^32,40^ The rate constants *k*_app_ was calculated to be 13.98 × 10^−3^ s^−1^ from the slope of the plots (Fig. S7†). Another significant parameter turnover frequency (TOF), defined as moles of reactant reduced through per mole of catalyst per second, was calculated to be 78 h^−1^. The comparison of the kinetic parameter of sodium sesquicarbonate-supporting Fe_2_O_3_@Pt with that of previous work was shown in Table S1.†

To further verify the synergistic effect between Fe and Pt, the catalytic effects of sodium sesquicarbonate-supporting Fe_2_O_3_@Pt and sodium sesquicarbonate-supporting Pt and Fe_2_O_3_ alone on 4-NP reduction were compared. It can be seen from Fig. S8† that the catalytic effect of sodium sesquicarbonate-supporting Fe_2_O_3_@Pt is much better than that of sodium sesquicarbonate-supporting Pt and Fe_2_O_3_ alone. And the conversion of 4-NP was up to 90% in 4 min (Fig. S9†), which is much better than loading Pt and Fe separately. Therefore, the introduction of non-metallic Fe can not only reduce the consumption of precious metal Pt, but also improve the catalytic efficiency due to the synergistic effect.

In conclusion, the prepared sodium sesquicarbonate structure can not only prevent the aggregation of Pt and Fe_2_O_3_, but also ensure the interaction between Pt and Fe, and improve the catalytic activity for 4-NP reduction. The material could be used in heterogeneous catalysis, water treatment and green chemistry.

## Conclusions

In summary, the sodium sesquicarbonate-supporting Fe_2_O_3_@Pt with interconnected one-dimensional network structure is developed *via* etching Fe_3_O_4_@Pt@SiO_2_. The supporting role of sodium sesquicarbonate is not only prevent the aggregation of pt clusters and Fe_2_O_3_ nanoparticles, but also provide sufficient channels for mass transfer. At the same time, due to the synergistic effect between Pt and Fe, sodium sesquicarbonate-supporting Fe_2_O_3_@Pt shows good catalytic activity for 4-NP reduction. Moreover, the introduction of non-metallic Fe can not only reduce the consumption of precious metal Pt, but also improve the catalytic efficiency due to the synergistic effect. In conclusion, we believe the one-dimensional sodium sesquicarbonate-supporting Fe_2_O_3_@Pt will be attractive for broad applications in heterogeneous catalysis, water treatment and green chemistry.

## Author contributions

Xia Xu: methodology, writing – original draft. Liming Yang: characterization of the TEM and collected the data performed the analysis. Mingqiang Li: synthesis nanomaterials collected the data. Bing Hu: writing – review & editing, funding acquisition. Xia Xu: designed the experiment and wrote the revised the paper.

## Conflicts of interest

There are no conflicts to declare

## Supplementary Material

RA-013-D3RA01930F-s001
