# Supplementary material for: Remarkably and stable catalytic activity in reduction of 4-nitrophenol by sodium sesquicarbonate-supporting Fe2O3@Pt
Source: RSC Adv. 2023 May 3;13(20):13556–63. doi: 10.1039/d3ra01930f (PMC10155080; doi:10.1039/d3ra01930f)
Supplement: RA-013-D3RA01930F-s001 [file RA-013-D3RA01930F-s001.pdf]

Electronic Supplementary Material (ESI) for RSC Advances.
This journal is © The Royal Society of Chemistry 2023

Supporting Information

Remarkably and stable catalytic activity in reduction of 4-nitrophenol by Sodium sesquicarbonate-supporting Fe_2O_3@Pt

Xia Xu,[a] Mingqiang Li,[b] Liming Yang[a] and Bing Hu[a]*

[a] College of Science, Gansu Agricultural University, Lanzhou 730070, P. R. China.

[b] College of Chemistry, Xinjiang University, Urumqi, Xinjiang 830046, P. R. China

E-mail: xuxia@gsau.edu.cn (Dr. X. Xu)

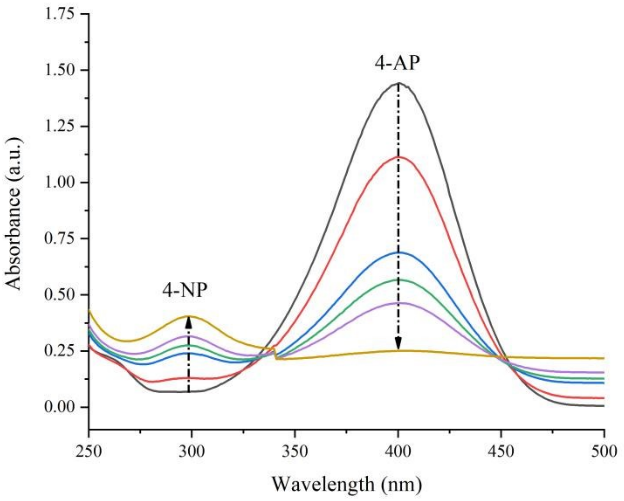

Fig. S1 Time-dependent UV-Vis spectra of the catalytic reduction of 4-NP catalyzed by Pt nanoparticles.

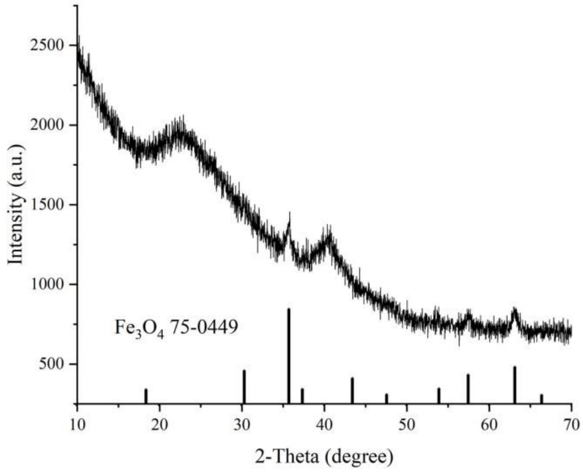

Fig. S2 X-ray powder diffraction (XRD) patterns of Fe_3O_4@Pt.

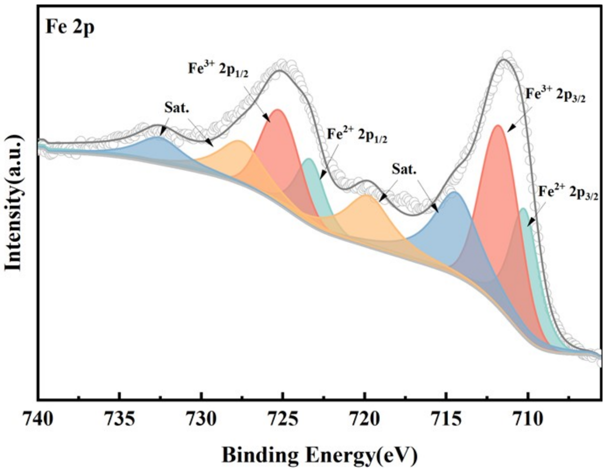

Fig. S3 **t**he Fe 2p XPS fine spectra of Fe_3O_4@Pt.

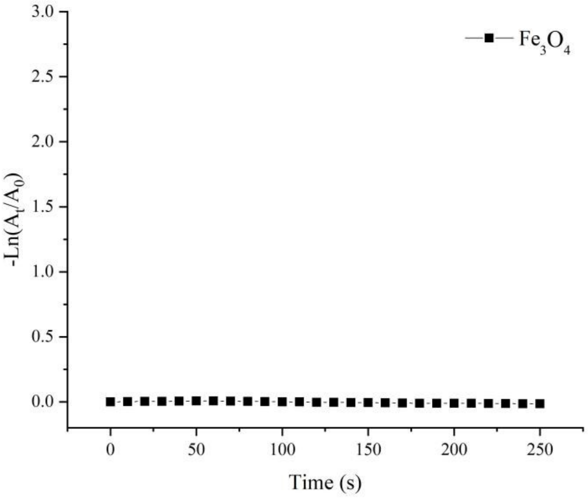

Fig. S4 Plots of -ln(A_t/A_0) versus the reaction time t for the reduction of 4-NP

catalyzed by Fe_3O_4.

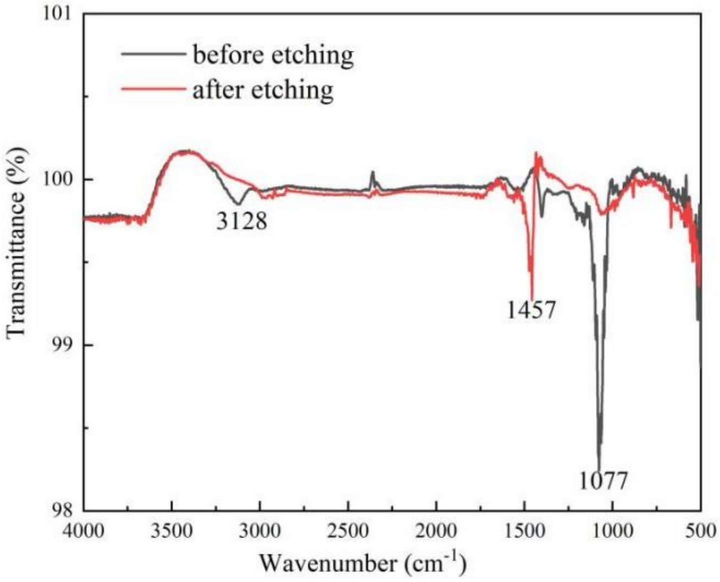

Fig. S5 FT-IR spectra of Fe_3O_4@Pt@SiO_2 (before etching) and sodium sesquicarbonate-supporting Fe_2O_3@Pt (after etching).

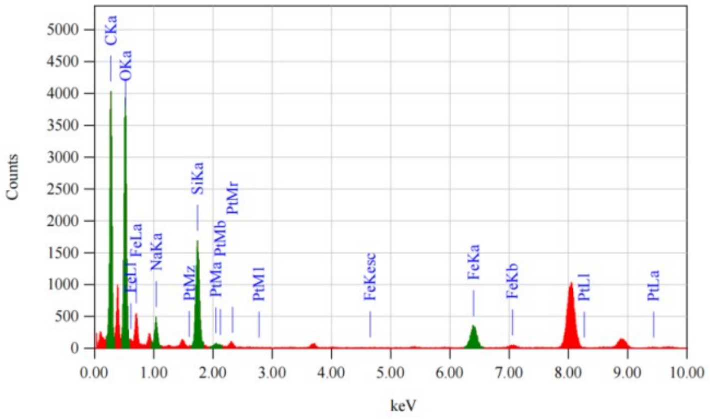

Fig. S6 EDX spectrum of sodium sesquicarbonate-supporting Fe_2O_3@Pt. The red peak can be attributed to the copper introduced by the copper network.

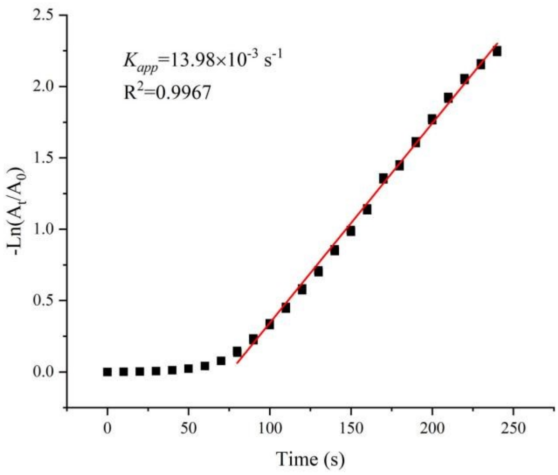

Fig. S7 Plots of -ln(A_t/A_0) versus the reaction time t for the reduction of 4-NP catalyzed by sodium sesquicarbonate-supporting Fe_2O_3@Pt.

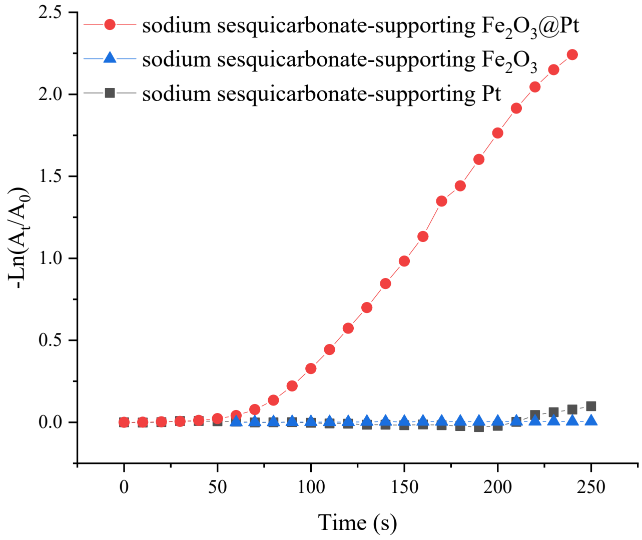

Fig. S8 Plots of -ln(A_t/A_0) versus the reaction time t for the reduction of 4-NP catalyzed by sodium sesquicarbonate-supporting Fe_2O_3@Pt and sodium sesquicarbonate-supporting Fe_2O_3 and Pt alone.

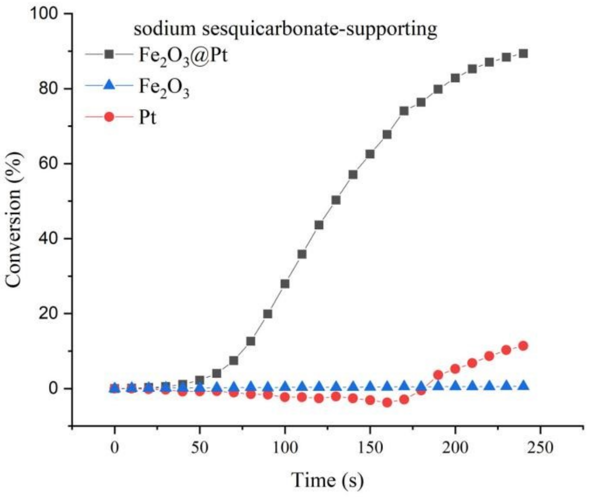

Fig. S9 The conversion versus the reaction time *t* for the reduction of 4-NPcatalyzed by sodium sesquicarbonate-supporting Fe_2O_3@Pt and sodium sesquicarbonate-supporting Fe_2O_3 and Pt alone.

Table S1 Comparison of the kinetic parameter of sodium sesquicarbonate-supporting Fe_2O_3@Pt for the reduction of 4-NP with that of previous work

Catalyst	Supporting materials	Reaction time	4-NP concentration (mM)	Catalyst concentration	Rate constant (10^{-3} s^{-1})	TOF /h^{-1}	Ref
AuNPs-glucan bioconjugates	Glucan (Pleurotus florida)	-	2	0.5 mM	0.33	-	1
MBS-AuNPs	Starch	13 min	1	-	0.33	-	2
AgNPs@MWCNTspolymer	Chitosan composite	5 min	0.1	10 mg	7.8	-	3
Pd/RGO/Fe_3O_4	Withania coagulans leaf	60 min	2.5	5 mg	51	-	4
Pd-GA/RGO	Gum arabic	5 min	5	1-20 mg	2	-	5
Au@graphitic	Carbon nitride nanocomposites	1 min	10	2 mg	15	-	6
AgNPs/ SiNSs	NPs/ SiNSs	-	0.12	0.051 mM	80.19	200	7
Cu/Pd@graphitic carbon	graphitic carbon	-	0.05	0.083 mM	80	108	8
PtAu-PDA/RGO	PDA/RGO	-	0.1	0.01 mM	9.58	200	9
sodium sesquicarbonate-supporting Fe_2O_3@Pt	sodium sesquicarbonate	4 min	0.067	0.013 mM	13.98	78	This works

Reference

1. I. K. Sen, K. Maity and S. S. Islam, Green synthesis of gold nanoparticles using a glucan of an edible mushroom and study of catalytic activity. *Carbohydr. Polym.*, 2013, **91**, 518.

2. S. Chairam, W. Konkamdee and R. Parakhun, Starch-supported gold nanoparticles and their use in 4-nitrophenol reduction. *J. Saudi Chem. Soc.*, 2015, **6**, 656.

3. S. M. Alshehri, T. Almuqati, N. Almuqati, E. Al-Farraj, N. Alhokbany and T. Ahamad, Chitosan based polymer matrix with silver nanoparticles decorated multiwalled carbon nanotubes for catalytic reduction of 4-nitrophenol. *Carbohydr. Polym.*, 2016, **151**, 135.

4. M. Atarod, M. Nasrollahzadeh and S. M. Sajadi, Green synthesis of Pd/RGO/Fe_3O_4 nanocomposite using Withania coagulans leaf extract and its application as magnetically separable and reusable catalyst for the reduction of 4-nitrophenol. *J. Colloid Interface Sci.*, 2016, **465**, 249.

5. A. Vilian, S. R. Choe, K. Giribabu, S. Jang, C. Roh, Y. S. Huh and Y. Han, Pd nanospheres decorated reduced graphene oxide with multi-functions: Highly efficient catalytic reduction and ultrasensitive sensing of hazardous 4-nitrophenol pollutant. *J. Hazard. Mater.*, 2017, **5**, 54.

6. T. B. Nguyen, C. P. Huang and R. A. Doong, Enhanced catalytic reduction of nitrophenols by sodium borohydride over highly recyclable Au@graphitic carbon nitride nanocomposites. *Appl. Catal., B*, 2019, **240**, 337.

7. W. Ye, J. Yu, Y. Zhou, D. Gao, D. Wang, C. Wang and D. Xue, Green synthesis of Pt-Au dendrimer-like nanoparticles supported on polydopamine-functionalized graphene and their high performance toward 4-nitrophenol reduction. *Appl. Catal., B*, 2016, **181**, 371.

8. Z. Yan, L. Fu, X. Zuo and H. Yang, Green assembly of stable and uniform silver nanoparticles on 2D silica nanosheets for catalytic reduction of 4-nitrophenol. *Appl. Catal., B*, 2018, **226**, 23.

9. M. Morales, M. Rocha, C. Freire, E. Asedegbega-Nieto, E. Gallegos-Suarez, I. Rodríguez-Ramos and A. Guerrero-Ruiz, Development of highly efficient Cu versus Pd catalysts supported on graphitic carbon materials for the reduction of 4-nitrophenol to 4-aminophenol at room temperature. *Carbon*, 2017, **111**, 150.
